# Age, but not hand preference, is related to personality traits in common marmosets (*Callithrix jacchus*)

**DOI:** 10.1098/rsos.220797

**Published:** 2022-10-19

**Authors:** Michaela Masilkova, Vedrana Šlipogor, Guilherme Henrique Lima Marques Silva, Magdaléna Hadová, Stanislav Lhota, Thomas Bugnyar, Martina Konečná

**Affiliations:** ^1^ Department of Game Management and Wildlife Biology, Faculty of Forestry and Wood Sciences, Czech University of Life Sciences, Prague, Czech Republic; ^2^ Department of Animal Science and Food Processing, Faculty of Tropical AgriSciences, Czech University of Life Sciences, Prague, Czech Republic; ^3^ Department of Zoology, Faculty of Science, University of South Bohemia, České Budějovice, Czech Republic; ^4^ Department of Behavioral and Cognitive Biology, Faculty of Life Sciences, University of Vienna, Vienna, Austria; ^5^ Bratislava Zoo, Bratislava, Slovakia; ^6^ Ústí nad Labem Zoo, Ústí nad Labem, Czech Republic

**Keywords:** behaviour coding, temperament, cerebral lateralization, handedness, laterality

## Abstract

The proximate mechanisms underlying animal personalities, i.e. consistent inter-individual differences in behaviour, are a matter of discussion. Brain lateralization, expressed as the preferred use of the contralateral limb, has been suggested as one of these mechanisms. In this study, we measured a proxy of brain lateralization in captive common marmosets (*N* = 28) by testing hand preference in a simple food-reaching task and evaluated personality by coding a wide range of behaviours observed in daily situations. We explored the links between personality and both direction and strength of hand preference, as well as age and sex, using linear models. Principal component analysis revealed that the stable behavioural variables were organized in three personality dimensions: Agreeableness, Extraversion and Neuroticism. Regarding hand preference, 14 individuals were left-handed, seven were right-handed and seven were ambilateral. Contrary to our predictions, we did not find any relationship between personality scores and hand preference or sex. Instead, age was a significant predictor of personality scores, with older individuals being more agreeable and less extraverted. The link between brain lateralization and personality seems to be equivocal and dependent on personality and brain lateralization assessment methods. Further examinations of other proximate mechanisms, such as physiology or (epi)genetics, may elucidate what drives personality variation in common marmosets.

## 1. Introduction

The research on animal personality, i.e. on consistent inter-individual differences in behaviour, has gained increasing attention in behavioural and cognitive biology in recent years [1,2]. Animal personality assumes both between-individual variation and within-individual consistency [3]. However, our understanding of its underlying proximate mechanisms is still quite limited [4,5]; while some of the personality traits seem to have a genetic or epigenetic basis [6,7], other personality traits may be governed by physiological, neuroendocrine, morphological or ontogenetic mechanisms [8–11].

Brain lateralization, i.e. asymmetry in hemispheric functions, has been hypothesized as one of the possible proximate neurophysiological mechanisms that maintain personality variation [12,13]. Brain lateralization is expressed as a preference for using the contralateral part of the body and is often measured either as a limb preference [14–18], eye dominance [19,20] or tail and body orientation [12,21,22]. Brain lateralization is present both in vertebrates and invertebrates (reviewed in [23,24]), and it provides various benefits in terms of enhanced cognition, social cohesion and better predator detection while foraging [25–27], thus directly affecting an individual's survival. Yet, population-level handedness (i.e. the tendency of the majority of the population toward using a specific hand as is known in humans) is extremely rare in animals [17]. Instead, vast individual variation in direction (i.e. preference for the left or right side of the body) and strength (i.e. magnitude of the preference irrespective of its direction) of lateralization exists within populations [28] and has been proposed to be linked with personality [13].

According to the ‘approach-withdrawal hypothesis’ [29], each hemisphere is specialized in processing and activating different types of behaviours. The left hemisphere controls approach-related behaviour, while the right hemisphere controls withdrawal behaviour. This general pattern has been confirmed across species and behaviours [14,30]. Individuals with right body side preference (i.e. left hemisphere dominance) are generally more explorative and bold [12] and are faster in approaching and inspecting novel objects [31]. These individuals also differ in their social interactions [28]. For example, pigs with right lateral bias were generally more sociable than their left lateral bias counterparts [12]. Right-pawed cats scored higher on playfulness [32]. Similarly, right-handed rhesus macaques received more grooming and spent more time in proximity to others than left-handed macaques, who were more frequently attacked and exhibited more submissive behaviours [33].

Personality has also been attributed to the strength of lateralization. Dogs and primates with stronger lateralization were more aggressive than their ambilateral (i.e. individuals without side preference) counterparts [31,34]. Similarly, humans with strong hand preference were rated as more extroverted than ambilateral humans, who were rated as more introverted [35]. On the other hand, ambilateral dogs tended to be more sociable, playful and bold [15].

The link between brain lateralization and personality might be species-, task- or trait-specific. For example, there was no correlation between the swimming direction and stress reactivity or docility in Port Jackson sharks [22,36]. Likewise, studies reported no association between limb preference and scores on personality dimensions in cats [32], dogs [34] or donkeys [16]. On an item level, however, limb preference significantly predicted the item ‘difficult to handle’ in donkeys [16] and ‘playful’ in cats [32]. The association of lateral bias and personality might also interact with other factors, such as sex (e.g. rhesus macaques [33,37] and humans [38], but see [35]). In cichlid fish, males with left eye dominance were more aggressive than males with right eye dominance. However, the opposite relationship was found in females [39]. Studies on humans did not report any clear pattern, and the link between lateral bias and personality seems to be equivocal [40]. While some studies confirmed personality differences between left- and right-handed individuals along the approach-withdrawal hypothesis [40,41], others found a reversed relationship [38,40] or no evidence for the link with personality [35,42]. Thus, studies on non-human primates could contribute to understanding the evolutionary mechanisms underlying the complex relationship between lateral bias and personality. Here, both aspects of laterality, strength and direction, should be investigated as potential underpinnings of consistent individual variation in behaviour.

Brain lateralization expressed as hand preference has been already studied in common marmosets (*Callithrix jacchus*) [43–46], New World primates, that are due to their size and ease of breeding one of the most common primate species in the laboratory [47,48]. Hand preference develops early in marmosets' life (5–8 months) and strengthens with time, stabilizing after the second year of life [49]. Hand preference in marmosets correlates with other motor preferences like mouth use [44] but not with sensory preferences [44,50]. Studies on marmosets found no population-level bias, yet they report vast individual variation in hand preference [44,45,49–53, cf. 54]. This individual variation is not, however, related to sex or age differences [46,51–53,55], but is likely due to other factors, like body posture and visual demands of the task [43,49,52], relatedness or social facilitation [49,54], or basal cortisol levels [53].

Behavioural studies of common marmosets confirmed the link between hand preference and reactions to novel or threatening stimuli in experimental settings [30,46,56,57]. Across studies, right-handed marmosets exhibited more explorative, active, approach-prone and less fearful behaviours than the left-handed marmosets (reviewed in [30]). Specifically, right-handed marmosets touched more objects, were faster to enter a novel room or to approach novel food [46], emitted more vocalizations when confronted with novel food [57], reacted to predator vocalizations with less freezing behaviour [56], and exhibited more social behaviours [53]. However, none of the mentioned studies directly tested the relationship between hand preference and personality measured as repeated behavioural reactions, on a broader construct level.

Personality of common marmosets has already been assessed with a variety of methods, including trait rating as well as experimental and common behaviour coding [58–62]. The resulting personality structures show considerable cross-method and ecological validity [63,64], and individual personality scores exhibit short- and long-term stability [59,60,63]. To our knowledge, only one captive study has assessed the potential links between hand preference and personality in common marmosets so far [55]. Combining experiments and common behaviour coding, a single broad personality dimension labelled as Inquisitiveness was revealed, describing individuals’ interest in food, novelty and friendliness to conspecifics. Inquisitiveness was not related to the direction of hand preference, but rather to the strength of hand preference, with more strongly lateralized individuals being more inquisitive than weakly lateralized individuals [55]. To fully understand the complex associations between brain lateralization and personality, it is necessary to explore the hand preference and its links with a broad range of personality dimensions (i.e. other than just Inquisitiveness).

Thus, the main objective of this study was to investigate the relationship between personality traits, measured by common behaviour coding, and brain lateralization, measured as hand preference in a simple food-reaching task, in captive common marmosets. We expected that the marmosets will show consistent differences in their hand use and that the handedness will be related to their personality type. Specifically, we predicted that right-handed individuals will be more extraverted, agreeable and assertive than left-handed individuals [33,55,59]. We also predicted that more strongly lateralized individuals will be more extraverted than weakly lateralized individuals [55].

## 2. Material and methods

### 2.1. Subjects

The subjects of this study were common marmosets housed in the Animal Care Facility of the Department of Behavioural and Cognitive Biology, University of Vienna, Austria. Marmosets were housed in social groups in indoor-outdoor enclosures equipped with branches, shelves, sleeping baskets and enrichment that was frequently changed. They were fed twice a day, in the morning with New World monkey pellets and around lunchtime with a varied diet consisting of fruits, vegetables, yoghurt, marmoset gum and jelly, and protein sources like insects or cooked chicken. Water was available ad libitum. More information on animal husbandry can be found in Šlipogor *et al.* [63].

### 2.2. Personality assessment

We assessed personality through common behaviour coding [65], which refers to the recording of the behaviour of animals in their social groups during everyday situations. In total, 33 marmosets older than 12 months (F = 12, M = 21) were observed in three study periods: in 2015 (*n* = 17), in 2016 (*n* = 7) and in 2020/2021 (*n* = 9) (see electronic supplementary material, table S1). In 2015 and 2016, each marmoset was followed for a total of 10 h (i.e. 240 h in total) over a period of 8 to 31 days. In 2020/2021, we collected 7 h of observation per individual instead (i.e. 63 h in total) over a period of 25 to 62 days (for details, see electronic supplementary material, table S1), because 5 to 7 h were found to be sufficient to capture personality in another callitrichid species [65].

In 2015 and 2016, MM used a voice recorder (Olympus VN-8700PC Digital Voice Recorder) to capture the behavioural observations, and afterward transcribed them into Microsoft Excel (Microsoft Office Professional Plus 2019). In 2020/2021, the behaviour was filmed by GM with a video camera (Canon LEGRIA HF R806), and the recordings were afterwards coded from a video player (Kakao PotPlayer v. 2002004 1.7.21126; 5KPlayer v. 6.8) into Microsoft Excel by GM (84 videos) and MM (42 videos). Before starting observations and coding videos, MM trained GM to ensure sufficient inter-rater reliability.

We used a combination of focal continuous and instantaneous sampling [66]. The focal period lasted 30 min with a sampling interval each 2 min. The focal periods for each individual were equally distributed during the day (from 7:30 am to 5:00 pm). We observed a wide range of behaviours as defined in a previously published ethogram [59] (for details see electronic supplementary material, table S2). From these, we calculated 22 behavioural indices following Masilkova *et al.* [59] (electronic supplementary material, table S3). They comprised three types of variables: frequencies e.g. *Scratching^F^*—frequency of scratching per hour), proportions of time (e.g. *Affiliation^P^*—the proportion of time focal subject spent in affiliative behaviours such as contact, proximity, social play and allogrooming), and diversity indices (e.g. *Activity diversity^S^*—Shannon diversity index of activity types; Shannon diversity index, computed according to Shannon & Weaver [67], describes the variation in behavioural diversity, low value = no behavioural diversity, high value = high behavioural diversity). Because of the different nature of indices (i.e. frequencies, proportions and Shannon diversity indices) and their range of possible values, we transformed the indices into z-scores (i.e. re-scaled them, so the mean value of index = 0 and s.d. = 1).

### 2.3. Hand preference test

**Figure 1.**
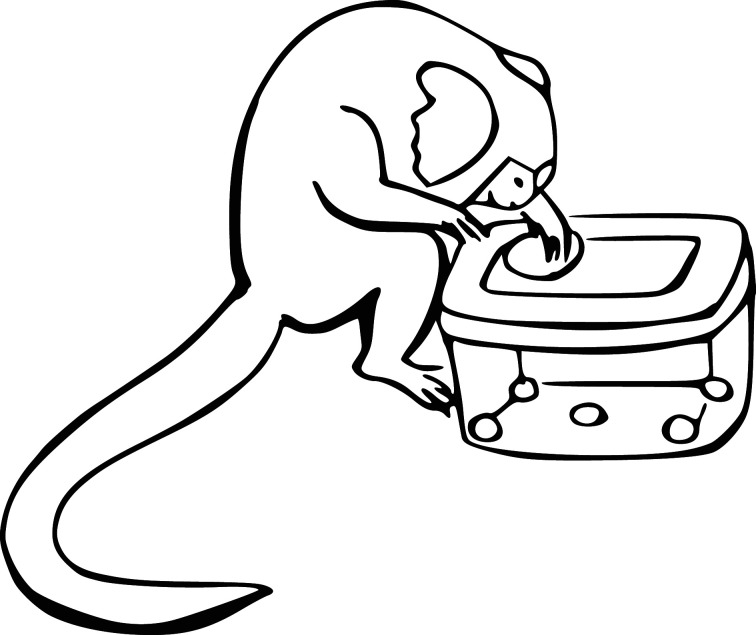
A marmoset performing a food-reaching task. Drawn by VŠ.

We assessed hand preference using a simple food-reaching task in 28 marmosets (F = 10, M = 18) in 2019 and 2021. Marmosets were tested individually in the experimental cage (72 *×* 42 *×* 110 cm). For the food-reaching task, we used a closed semi-transparent plastic box (9 *×* 9 cm) with a hole in one of the lid's corners (diameter: 2.5 cm). The box contained five small pieces of banana (diameter: 1 cm): four placed in the corners and one placed in the middle of the box. We noted the number of times and which hand an individual used to access a piece of food through the hole (figure 1). Tests lasted until the marmosets collected all five banana pieces, and not more than 8 min. The experiment was recorded with a video camera (Canon LEGRIA HF R806). The experimental enclosure was thoroughly cleaned with a vinegar–water solution between different individuals.

Each individual was tested between 2 and 6 times (i.e. 10*–*30 hand preferences scores, depending on the age-criterion and availability in different testing periods) in two different periods (two trials in March 2019 by MH, four trials in February 2021 by GM, see electronic supplementary material, table S1) and with a minimum interval of 48 h between each trial. We calculated the direction and strength of the hand preference only once all the behavioural observations were done to prevent any possible involuntary influences on the personality assessments.

### 2.4. Data analyses

All analyses were conducted in R studio (v. 1.2.5019 [68]). To control for inter-rater reliability, a subset of videos from 2020/2021 (10%) was coded by both observers (GM and MM) and the Intra-Class Correlation Coefficients (3,1; two-way, agreement, single) and associated 95% confidence intervals were computed in package *irr* [69], independently for substrate and behaviour instances in the instantaneous sampling, and behaviour instances in the continuous recording.

Subsequently, we calculated the repeatability estimates of the behavioural indices, which reflect the proportion of behavioural variation attributed to differences between individuals [70]. Because the overall length and method of observation differed between the first two and the third study periods, we assessed the repeatability of behavioural indices for the first two study periods, for which we had the same observation length (*n* = 24). We split the observations into two 5-h blocks per individual and computed the repeatability between them. The repeatability was calculated using linear mixed models with an individual as a random factor in package *rptR* [71]. The *p* values and 95% confidence intervals were calculated from 1000 permutation and 1000 bootstrap runs [70].

Only significantly repeatable behavioural indices entered the personality analyses. We checked the sampling adequacy using the Keiser–Meyer–Olkin test (KMO) and Bartlett's Test of Sphericity, both as functions of package *psych* [72]. To determine the number of personality dimensions in the data, we ran Horn's parallel analysis [73] and inspected scree plots [74] using package *paran* [75]. We then conducted the Principal Component Analysis (PCA) in package *psych* [72] to identify the personality components. Next, we rotated the resulting component structure using oblique (Promax) and orthogonal (Varimax) rotations, the former enabling intercorrelations among components. We interpreted the Varimax rotated structure if the correlations were negligible and the resulting structures similar. Loadings ≥ |0.4| were considered salient [58,76,77].

After that, we computed a unit-weighted personality score for each marmoset on each resulting personality component. The unit-weighted scores are the sum of all indices that loaded saliently on a dimension. Indices that loaded positively are weighed by plus one, and indices that loaded negatively are weighed by minus one [78].

The direction of hand preference was calculated with the following formula: HI = (*R*−*L*)/*N*. Here, ‘HI’ stands for the handedness index, ‘R’ for the number of right-hand instances, ‘L’ for the number of left-hand instances and ‘N’ for the total number of instances [56]. HI ranges from −1 to +1. Zero on the index indicates no hand preference, a positive index value indicates a right-hand preference and a negative index value signifies a left-hand preference. To obtain a statistically significant indication of hand preference, we calculated *z*-score using the formula: *z* = (*R*−0.5N)/√(0.25N) (for abbreviations, see above). The values of *z* > 1.96 were considered the statistically significant indication of the right-hand preference, *z* < −1.96 the indication of the left-hand preference, and values between −1.96 and 1.96 the indication of no-hand preference [57]. In addition to direction, we calculated the strength of hand preference as an absolute value of HI (hereafter absHI). The absHI ranges from 0 (no or weak hand preference) to 1 (strong hand preference regardless the side).

To further explore the hand preference in common marmosets and to assess the consistency of HI between the two study periods (2019 versus 2021), we calculated the Spearman rank-order correlations. Two linear models were run with age (in months) and sex (M, F) as fixed factors and HI or absHI as a response to assess the effect of age and sex on handedness measures. Finally, we used the Chi-Squared test to determine the bias in the distribution of left, right and ambilateral preferences at the population level.

Linear models were used to assess the relationship between personality scores, direction and strength of hand preference, sex and age. The appropriateness of model parameters was visually checked using *plot* function, and the collinearity of the predictors was checked using variance inflation factors (*vif* function, all less than 1.29). We ran three separate models for each personality dimension (Agreeableness, Extraversion, Neuroticism) with HI, absHI, age (at the time of personality data collection, in months) and sex (M, F) as fixed predictors.

## 3. Results

### 3.1. Personality

The inter-observer reliability of personality observations was excellent for substrate types (ICC (3,1) = 0.985, 95% CI [0.976, 0.991]) and behaviour instances (ICC (3,1) = 0.927, 95% CI [0.897, 0.949]) in instantaneous sampling and behaviour instances (ICC (3,1) = 0.969, 95% CI [0.957, 0.978]) in continuous recording. In terms of repeatability of behavioural measures, from 22 behavioural indices, only three were not repeatable across two time points within 2015 and 2016 study periods: *Activity diversity^S^* (*R* = 0.24, *p* = 0.13), *Carrying food away^F^* (*R* = 0.34, *p* = 0.05) and *Invite grooming(rec)^F^* (*R* = 0.19, *p* = 0.21). The remaining 19 indices were repeatable (mean *R* ± s.d: 0.68 ± 0.17) and thus were considered for further personality analyses. Among repeatable indices, *Threats^F^* had the highest repeatability (*R* = 0.95). Two indices (*Resting^P^* and *Passive affiliation^P^*) with the lowest repeatabilities (*R* = 0.34 and *R* = 0.40, respectively) contained zero in the confidence interval but we nevertheless considered them repeatable (due to *p* < 0.05). For details, table 1.

**Table 1.** The repeatability of behavioural indices across two five-hour long blocks of observation.

behavioural index	*R* ± SE	95% CI	*p*
*activity diversity^S^*	0.24 ± 0.17	[0, 0.58]	0.13
*affiliation^P^*	**0.71** ± 0.11	[0.44, 0.87]	0.001
*approaches^F^*	**0.61** ± 0.14	[0.29, 0.81]	0.001
*carrying food away^F^*	0.34 ± 0.17	[0, 0.62]	0.05
*contact aggression^F^*	**0.91** ± 0.04	[0.80, 0.96]	0.001
*departures^F^*	**0.68** ± 0.12	[0.41, 0.85]	0.002
*exploration^F^*	**0.83** ± 0.07	[0.67, 0.92]	0.001
*grooming(in)^F^*	**0.52** ± 0.15	[0.16, 0.76]	0.009
*grooming(rec)^F^*	**0.69** ± 0.12	[0.38, 0.85]	0.001
*invite grooming(in)^F^*	**0.61** ± 0.14	[0.28, 0.82]	0.007
*invite grooming(rec)^F^*	0.19 ± 0.16	[0, 0.54]	0.21
*monitoring^P^*	**0.62** ± 0.13	[0.32, 0.80]	0.002
*object sniffing^F^*	**0.79** ± 0.08	[0.58, 0.91]	0.001
*passive affiliation^P^*	**0.40** ± 0.18	[0, 0.70]	0.03
*resting^P^*	**0.35** ± 0.17	[0, 0.65]	0.04
*scent marking^F^*	**0.84** ± 0.07	[0.68, 0.93]	0.001
*scratching^F^*	**0.78** ± 0.09	[0.56, 0.90]	0.001
*self-grooming^F^*	**0.77** ± 0.09	[0.55, 0.88]	0.001
*substrate diversity^S^*	**0.51** ± 0.16	[0.14, 0.76]	0.005
*terminate grooming^F^*	**0.50** ± 0.15	[0.13, 0.74]	0.009
*threats^F^*	**0.95** ± 0.02	[0.89, 0.98]	0.001
*vigilance^F^*	**0.88** ± 0.05	[0.73, 0.94]	0.001

Note. *^F^* = frequency of behaviour per hour, *^P^* = proportion of time, *^S^* = Shannon diversity index [67]. The significantly repeatable indices are in bold.

The KMO measure of PCA sampling adequacy was 0.4, below the recommended level of 0.6 [79,80]*.* On the other hand, Bartlett's test of sphericity indicated that the sampling is adequate for PCA (*χ*^2^ = 459.05, d.f. = 171, *p* < 0.01) [79,81]. Horn's parallel analysis and visual inspection of the scree plot suggested retaining four components in the data. Because the Promax- and Varimax-rotated four-component structures did not differ considerably, and inter-component correlations were low, we interpreted the Varimax-rotated structure, which explained 61% of the variance (Promax solution in electronic supplementary material, table S4, Varimax solution in electronic supplementary material, table S5). Yet, its fourth component was difficult to interpret meaningfully.

Thus, we retained the three-component solution instead. Because Promax- and Varimax-rotated three-component solutions were almost identical (Promax solution in electronic supplementary material, table S6), we interpreted the Varimax solution, which explained 49% of the variance (table 2). The first component included positive loadings of grooming-related indices (e.g. *Grooming(in)^F^*) and negative loadings of aggression-related indices (e.g. *Contact aggression^F^*). Therefore, we labelled it as Agreeableness. The second component was defined by indices related to the social activity (e.g. *Approaches^F^*) and exploration (e.g. *Exploration^F^*). We thus named it Extraversion. The third component included positive loadings of indices such as *Scratching^F^*, *Vigilance^F^* and *Scent marking^F^*, so we labelled it as Neuroticism. From 19 repeatable indices, two indices (*Monitoring^P^* and *Resting^P^*) did not load saliently on any component.

**Table 2.** The Varimax-rotated principal component structure.

	agreeableness	extraversion	neuroticism	*h*^2^
*terminate grooming^F^*	**0.76**	0.01	−0.05	0.59
*grooming(in)^F^*	**0.74**	0.03	−0.07	0.55
*threats^F^*	***−*0.67**	0.30	0.19	0.58
*contact aggression^F^*	***−*0.58**	−0.05	−0.20	0.38
*grooming(rec)^F^*	**0.54**	−0.32	**0.41**	0.56
*monitoring^P^*	*−*0.39	−0.02	−0.02	0.16
*departures^F^*	*−*0.07	**0.79**	0.20	0.68
*approaches^F^*	*−*0.28	**0.76**	0.31	0.75
*exploration^F^*	*−*0.02	**0.73**	0.00	0.54
*passive affiliation^P^*	*−*0.27	***−*0.71**	−0.11	0.58
*affiliation^P^*	0.28	***−*0.49**	**−0.45**	0.52
*substrate diversity^S^*	**0.42**	**0.45**	−0.30	0.47
*resting^P^*	0.04	−0.29	0.20	0.13
*object sniffing^F^*	0.01	−0.02	**0.84**	0.71
*scent marking^F^*	*−*0.33	0.06	**0.73**	0.65
*invite grooming(in)^F^*	**0.42**	−0.03	**0.63**	0.58
*scratching^F^*	*−*0.16	0.18	**0.59**	0.41
*vigilance^F^*	0.09	−0.01	**0.45**	0.21
*self-grooming^F^*	0.12	0.26	**0.40**	0.24
explained variance	16%	17%	16%	

*N*ote. *^F^* = frequency of behaviour per hour, *^P^* = proportion of time, *^S^* = Shannon diversity index [67], *h^2^* = communalities. Salient loadings ≥ |0.4| in bold.

### 3.2. Hand preference

According to the HI *z*-score, 14 individuals (F = 7, M = 7) were left-handed, seven individuals (F = 2, M = 5) were right-handed and seven individuals (F = 1, M = 6) were ambilateral (electronic supplementary material, table S7). The direction of hand preference was consistent (Spearman's rank-order correlation: *r*_S_ = 0.94, *p* < 0.001) across two study periods (2019 versus 2021). There was no effect of age and sex on either the direction (all *p* > 0.18) or the strength (all *p* > 0.35) of hand preference. For the full results of the linear models, see electronic supplementary material, table S8. The observed distribution of hand preference did not significantly differ from that expected by chance (*χ*^2^ = 3.5, *p* = 0.17); hence, there was no population-level bias in hand preference in our study population.

### 3.3. The link between personality, hand preference, sex and age

**Figure 2.**
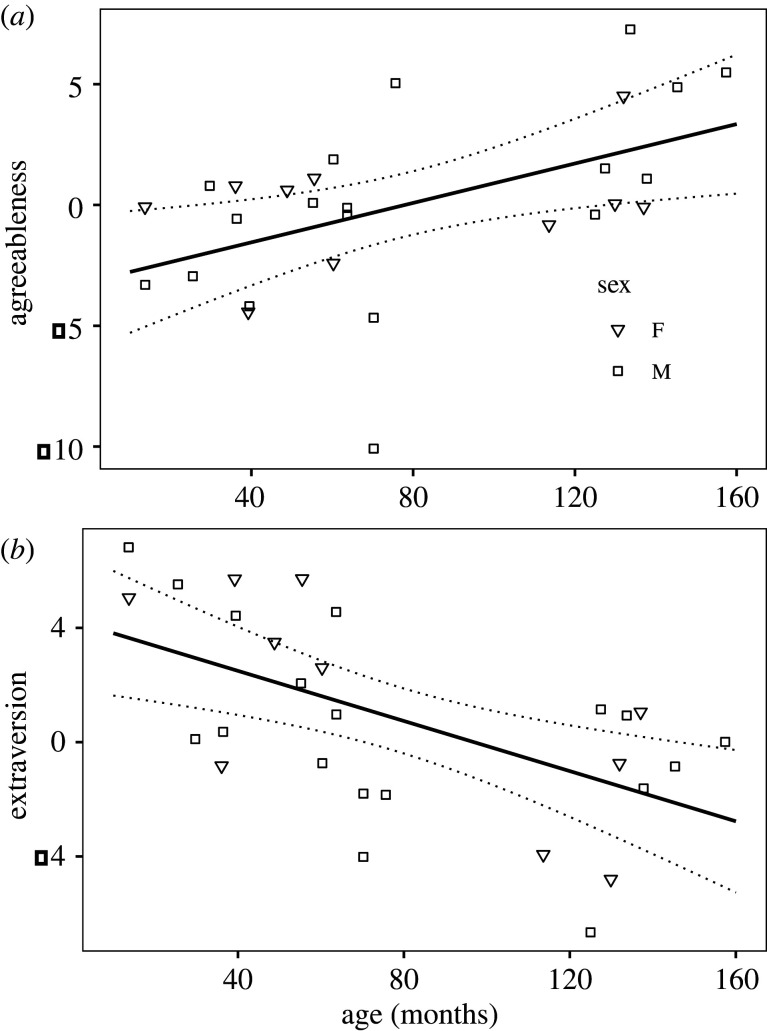
The relationship between age (in months) and Agreeableness (*a*) and Extraversion (*b*) personality z-scores from linear model. The area represented by dotted lines depicts 95% confidence interval.

Agreeableness was significantly related only to age, as older individuals had higher Agreeableness scores (slope mean ± SE = 0.041 ± 0.015, *p* = 0.013; figure 2*a*). The relationships with sex, absHI and HI were non-significant (all *p* > 0.88). The results were qualitatively similar for Extraversion, as older individuals had significantly lower scores on Extraversion (slope mean ± SE = −0.044 ± 0.013, *p* = 0.003; figure 2*b*), and the other three relationships were not significant (all *p* > 0.42). The Neuroticism scores were not significantly predicted by any of the four predictor variables (all *p* > 0.50; see electronic supplementary material, table S9 for details; see electronic supplementary material, figure S1 for the relationship between personality and HI and absHI).

## 4. Discussion

Personality structure in common marmosets, evaluated by behavioural observations in daily situations, comprised three personality dimensions (Agreeableness, Extraversion and Neuroticism). We also detected individual differences in right-, left- and ambilateral hand preference in a simple food-reaching task. Contrary to our predictions, neither direction nor strength of hand preference were linked to individual scores on personality dimensions. The personality variation was instead explained by age differences, with older individuals being more agreeable and less extraverted than younger ones.

The resulting personality dimensions were broadly comparable to the personality dimensions previously described in common marmosets using the same [62] as well as different methods of personality evaluation such as trait rating [48,58,62,82,83] and experimental coding [60,63,64]. The personality structure was also comparable but not completely identical to the structure reported in our previous study based on the same method of personality assessment and a subset of individuals (*N* = 17) [59]. Therefore, the observed individual behavioural variation reflected well the existing personality variation in this species.

Agreeableness, defined by high levels of grooming and low levels of aggression, resembles Sociability [58,83] and Agreeableness [62,82] assessed by trait rating and characterized by adjectives such as affectionate, sociable and friendly. Moreover, Agreeableness is similar to Agreeableness reported in our previous study [59] evaluated by common behaviour coding. Extraversion, depicting the exploration and physical activity of individuals, broadly resembles questionnaire-derived Openness [58,62] and Inquisitiveness [82] and experimentally derived Exploration-Avoidance [60]. In addition, Extraversion in our study is also defined by indices related to social activity, such as *Departures^F^* and *Approaches^F^*, which might, to some extent, reflect the general level of an individual's activity. Compared to our previous study [59], Extraversion was more narrowly specified. Neuroticism, defined by high levels of vigilance, scratching and self-grooming, resembles Neuroticism in the study using the same method of personality evaluation [62]. It also broadly resembles the questionnaire-derived Impulsiveness dimension [58] and Conscientiousness [82] that loaded items such as irritable or excitable. In our previous study [59], we detected Assertiveness that had positive loadings of *Threats^F^*, *Scent marking^F^* and *Invite grooming(in)^F^*—behaviours indicative of dominance [84]. Yet, the dimension in the current study did not load *Threats^F^* but had positive loadings of *Vigilance^F^* and *Self-grooming^F^.* In other words, an individual scoring high on this dimension exhibits frequent scratching, self-grooming, scent-marking and vigilant behaviour. These changes in the item content led us to the decision to label this dimension Neuroticism instead of Assertiveness. Indeed, several studies have confirmed the connection between scent marking, scratching, and stress in common marmosets [85,86].

In our study population, age was a strong predictor of Agreeableness and Extraversion personality scores (cf. [60]). In particular, older individuals were more agreeable and less extraverted than younger ones which corresponds to previous finding in the same species [82]. Nonetheless, the positive relationship between age and Agreeableness is surprising, as in white-faced capuchins, another New-World monkey species, the directionality of the link was reversed so that the older individuals were less agreeable [77]. Our results thus more resemble the results in chimpanzees and humans [87–89]. A possible explanation for the similar trend in humans and marmosets might be their socio-ecologies, particularly similarities in terms of cooperative breeding and living in family units (e.g. [47]). The negative relationship between age and Extraversion, i.e. that older individuals become less extraverted, reflects a general pattern found in New World monkeys and apes, including humans [77,87–90]. Studies on Old World monkeys, in contrast, reported positive relationship between age and Extraversion [91,92]. This similarity in age effect between our marmoset population and captive great ape populations is unexpected and perhaps driven by a particular composition of our individuals that included some quite old marmosets (mean = 78.4 mo), which is also often the case in the studied captive great ape populations.

Although some studies report sex-specific variation in primate personality (frequently in Dominance or Neuroticism related dimensions, e.g. [76,77,93]), we did not detect any sex effects on personality scores which is consistent with the general pattern found across animal taxa [94], and due to their cooperative breeding and biparental care of infants, should not be expected in common marmosets. Indeed, available marmoset personality studies either did not evaluate the sex effects [59,62–64,83,95] or did not find any statistically significant differences [60,61]. Koski *et al.* [82] found a significant interaction between sex and age in Agreeableness. We, however, could not test for this interaction due to the uneven distributions of individuals in the sex and age categories. To sum up, sex differences in personality in primates might have evolved independently in different species and might be connected to species' social or mating system [93].

In terms of hand preference, 50% of individuals were left-handed, 25% right-handed and 25% ambilateral. As in other studies on hand use in marmosets [49,51–53], we did not detect any population-level bias in hand preference. The individual hand preference was, however, highly consistent (across the time span of two years), but neither strength nor direction of hand preference were explained by individual's sex or age—a general pattern commonly reported for marmosets [51–53,55].

Although we detected stable individual differences in observed behaviour, this variation was not attributable to observed individual variation in hand use in marmosets. Contrary to our prediction, neither direction nor strength of hand preference in a food-reaching task were linked to personality, as found also in humans and other species [16,34,35,42]. For instance, Extraversion and Agreeableness were not associated with the direction of limb preference in donkeys [16], lateral bias did not correlate with docility in sharks [22], and most of the personality dimensions did not show a link with paw preference in dogs [34]. There are several possible explanations for this result. First, perhaps brain lateralization is not the proximate mechanism responsible for maintaining personality variation in the common marmoset and other species, including humans [35,42]. One of the possible suggested explanations for personality differences between left-handers and right-handers in humans stems from a different experience of left-handers as a minority group in a right-handed majority society. This experience includes possible negative prejudice and also difficulties while operating equipment designed for the right-handed population [41]. To sum up, other proximate mechanisms, such as genetics or hormone levels [83], may play a more significant role in the development of personality differences.

Second, even though Tomassetti *et al.* [55] reported more strongly lateralized marmosets being more inquisitive, we did not find a link between a similar personality dimension, Extraversion, and hand preference in our study. This is perhaps due to the different personality assessment methods used in these studies. Measuring personality differences as repeatable reactions to certain experimental stimuli, e.g. novel object or space, might target more specific dimensions related to approach–withdrawal reactions that are perhaps better reflected in brain lateralization, as demonstrated in common marmosets [55], pigs [12] or cichlids [19]. Trait rating and common behaviour coding, in contrast, yield broadly defined personality dimensions in which several seemingly not related behaviours might be correlated, such as social activity and tendency to explore (i.e. Extraversion in our study). As a result, these methods might not be sensitive enough to detect the positive association with brain lateralization [34]. This is further supported by the fact that trait rating studies failed to find a link between brain lateralization and individual scores on personality dimensions (e.g. impulsiveness). Instead, they report positive links between brain lateralization and single personality items (e.g. playful), such as, for instance, in donkeys and cats [16,32]. Future studies should, thus, use methods that describe narrowly specified personality traits.

Third, the preference for using one hand over the other in a particular task does not necessarily involve using a specific hemisphere and, as a result, may not reflect the general brain lateralization. Laterality is often viewed as a multifactorial phenomenon in which not all lateral biases are necessarily in the same direction, an issue frequently discussed also in the human literature [40]. In fact, in a study that compared the hand preference in a normal feeding situation and in a reaching task using an apparatus, almost half of the marmosets displayed opposite preferences in these two tasks [96]. The direction of hand preference was, however, retained when comparing hand preference during simple feeding and other tests. Therefore, the individual's universal hand preference may differ based on the complexity of the task, as well as their postural and visuospatial demands [52,97].

To conclude, even though there has been previous evidence that right-handed and left-handed marmosets behave differently [46,56], we did not find a link between hand preference and personality differences, assessed with behavioural observations. Instead, we detected a strong effect of age on personality scores, whereby older marmosets were more agreeable and less extraverted than the younger ones, which corresponds to the general pattern found in other primates. The association between hand preference and personality thus remains ambiguous. To tackle this ambiguity, future studies should employ methods targeting personality traits related to approach-withdrawal and tasks that can better reveal brain lateralization. Finally, it is possible that other proximate mechanisms, such as development, (epi)genetics or physiology, might instead underpin the personality variation in common marmosets, as well as other primate and mammalian species.

## Data Availability

The datasets and code supporting this article have been uploaded as part of the supplementary material [98].

## References

[1] Norman M, Rowden LJ, Cowlishaw G. 2021 Potential applications of personality assessments to the management of non-human primates: a review of 10 years of study. PeerJ **9**, e12044. (10.7717/peerj.12044) 34589296 PMC8432321

[2] Wilson V, Guenther A, Øverli Ø, Seltmann MW, Altschul D. 2019 Future directions for personality research: contributing new insights to the understanding of animal behavior. Animals **9**, 240. (10.3390/ani9050240) 31096599 PMC6562689

[3] Réale D, Reader SM, Sol D, McDougall PT, Dingemanse NJ. 2007 Integrating animal temperament within ecology and evolution. Biol. Rev. **82**, 291-318. (10.1111/j.1469-185X.2007.00010.x) 17437562

[4] Stamps J, Groothuis TGG. 2010 The development of animal personality: relevance, concepts and perspectives. Biol. Rev. Camb. Philos. Soc. **85**, 301-325. (10.1111/j.1469-185X.2009.00103.x) 19961473

[5] Trillmich F, Müller T, Müller C. 2018 Understanding the evolution of personality requires the study of mechanisms behind the development and life history of personality traits. Biol. Lett. **14**, 20170740. (10.1098/rsbl.2017.0740) 29491028 PMC5830666

[6] van Oers K, de Jong G, van Noordwijk A, Drent P. 2005 Contribution of genetics to the study of animal personalities: a review of case studies. Behaviour **142**, 1185-1206. (10.1163/156853905774539364)

[7] Dochtermann NA, Schwab T, Sih A. 2015 The contribution of additive genetic variation to personality variation: heritability of personality. Proc. R. Soc. B **282**, 20142201. (10.1098/rspb.2014.2201) PMC4262176 25392476

[8] Koolhaas JM, Korte SM, De Boer SF, Van Der Vegt BJ, Van Reenen CG, Hopster H, De Jong IC, Ruis MAW, Blokhuis HJ. 1999 Coping styles in animals: current status in behavior and stress- physiology. Neurosci. Biobehav. Rev. **23**, 925-935. (10.1016/S0149-7634(99)00026-3) 10580307

[9] Roulin A, Dreiss AN, Kölliker M. 2010 Evolutionary perspective on the interplay between family life, and parent and offspring personality. Ethology **116**, 787-796. (10.1111/j.1439-0310.2010.01793.x)

[10] Dammhahn M, Dingemanse NJ, Niemelä PT, Réale D. 2018 Pace-of-life syndromes: a framework for the adaptive integration of behaviour, physiology and life history. Behav. Ecol. Sociobiol. **72**, 62. (10.1007/s00265-018-2473-y)

[11] Hinde K, Capitanio JP. 2010 Lactational programming? Mother's milk energy predicts infant behavior and temperament in Rhesus Macaques (*Macaca mulatta*). Am. J. Primatol. **72**, 522-529. (10.1002/ajp.20806) 20162547 PMC3377500

[12] Goursot C, Düpjan S, Kanitz E, Tuchscherer A, Puppe B, Leliveld LMC. 2019 Assessing animal individuality: links between personality and laterality in pigs. Curr. Zool. **65**, 541-551. (10.1093/cz/zoy071) 31616485 PMC6784513

[13] Rogers LJ. 2009 Hand and paw preferences in relation to the lateralized brain. Phil. Trans. R. Soc. B **364**, 943-954. (10.1098/rstb.2008.0225) 19064357 PMC2666076

[14] Manns M, Basbasse YE, Freund N, Ocklenburg S. 2021 Paw preferences in mice and rats: meta-analysis. Neurosci. Biobehav. Rev. **127**, 593-606. (10.1016/j.neubiorev.2021.05.011) 34004244

[15] Barnard S, Wells DL, Hepper PG, Milligan ADS. 2017 Association between lateral bias and personality traits in the domestic dog (*Canis familiaris*). J. Comp. Psychol. **131**, 246-256. (10.1037/com0000074) 28414470

[16] Díaz S, Murray L, Rodway P. 2021 Laterality Limb preference and personality in donkeys (*Equus asinus*). Laterality Asymmetries Brain, Behav. Cogn. **26**, 186-200. (10.1080/1357650X.2021.1882480) 33545019

[17] Caspar KR, Pallasdies F, Mader L, Sartorelli H, Begall S. 2021 The evolution and biological correlates of hand preferences in anthropoid primates. bioRxiv Prepr. (10.1101/2021.12.30.474462) PMC9714969 36454207

[18] Kuběnová B, Lhota S, Tomanová V, Blažek V, Konečná M. 2022 Lion-tailed macaques show a stable direction and reinforcement of hand preference in simple reaching tasks over several years. J. Vertebr. Biol. **71**, 21076. (10.25225/jvb.21076)

[19] Reddon AR, Hurd PL. 2009 Individual differences in cerebral lateralization are associated with shy–bold variation in the convict cichlid. Anim. Behav. **77**, 189-193. (10.1016/j.anbehav.2008.09.026)

[20] Rogers LJ, Ward JP, Stanford D. 1994 Eye dominance in the small-eared bushbaby, *Otolemur garnettii*. Neuropsychologia **32**, 257-264. (10.1016/0028-3932(94)90011-6) 8190249

[21] Quaranta A, Siniscalchi M, Vallortigara G. 2007 Asymmetric tail-wagging responses by dogs to different emotive stimuli. Curr. Biol. **17**, 199-201. (10.1016/j.cub.2007.02.008) 17371755

[22] Byrnes EE, Pouca CV, Chambers SL. 2016 Into the wild: developing field tests to examine the link between elasmobranch personality and laterality. Behaviour **153**, 1777-1793. (10.1163/1568539X-00003373)

[23] Niven JE, Frasnelli E. 2018 Insights into the evolution of lateralization from the insects. In Progress in brain research, pp. 3-31. Amsterdam, The Netherlands: Elsevier B.V. 10.1016/bs.pbr.2018.06.001 30097197

[24] Vallortigara G, Bisazza A. 2002 How ancient is brain lateralization? In Comparative vertebrate lateralization (eds LJ Rogers, RJ Andrew), pp. 9-69. Cambridge, UK: Cambridge University Press.

[25] Piddington T, Rogers LJ. 2013 Strength of hand preference and dual task performance by common marmosets. Anim. Cogn. **16**, 127-135. (10.1007/s10071-012-0562-2) 23053795

[26] Rogers LJ, Zucca P, Vallortigara G. 2004 Advantages of having a lateralized brain. Proc. R. Soc. B **271**, 420-422. (10.1098/rsbl.2004.0200) PMC1810119 15801592

[27] Vallortigara G. 2006 The evolutionary psychology of left and right: costs and benefits of lateralization. Dev. Psychobiol. **48**, 418-427. (10.1002/dev.20166) 16886183

[28] Rogers LJ. 2021 Brain lateralization and cognitive capacity. Animals **11**, 1996. (10.3390/ani11071996) 34359124 PMC8300231

[29] Davidson RJ. 1992 Emotion and affective style: hemispheric substrates. Psychol. Sci. **3**, 39-43. (10.1111/j.1467-9280.1992.tb00254.x)

[30] Rogers LJ. 2018 Manual bias, behavior, and cognition in common marmosets and other primates. In Progress in brain research, pp. 91-113. Amsterdam, The Netherlands: Elsevier B.V. 10.1016/bs.pbr.2018.06.004 30097204

[31] Fernández-Lázaro G, Latorre R, Alonso-García E, Barja Núñez I. 2019 Nonhuman primate welfare: can there be a relationship between personality, lateralization and physiological indicators? Behav. Process. **166**, 103897. (10.1016/j.beproc.2019.103897) 31271769

[32] McDowell LJ, Wells DL, Hepper PG, Dempster M. 2016 Lateral bias and temperament in the domestic cat (*Felis silvestris*). J. Comp. Psychol. **130**, 313-320. (10.1037/com0000030) 27359075

[33] Westergaard GC, Chavanne TJ, Lussier ID, Houser L, Cleveland A, Suomi SJ, Higley JD. 2003 Left-handedness is correlated with CSF monoamine metabolite and plasma cortisol concentrations, and with impaired sociality, in free-ranging adult male rhesus macaques (*Macaca mulatta*). Laterality **8**, 169-187. (10.1080/713754484) 15513221

[34] Schneider LA, Delfabbro PH, Burns NR. 2013 Temperament and lateralization in the domestic dog (*Canis familiaris*). J. Vet. Behav. Clin. Appl. Res. **8**, 124-134. (10.1016/j.jveb.2012.06.004)

[35] Grimshaw GM, Wilson MS. 2013 A sinister plot? Facts, beliefs, and stereotypes about the left-handed personality. Laterality **18**, 135-151. (10.1080/1357650X.2011.631546) 23485059

[36] Byrnes EE, Vila-Pouca C, Brown C. 2016 Laterality strength is linked to stress reactivity in Port Jackson sharks (*Heterodontus portusjacksoni*). Behav. Brain Res. **305**, 239-246. (10.1016/j.bbr.2016.02.033) 26946274

[37] Westergaard GC, Chavanne TJ, Houser L, Cleveland A, Snoy PJ, Suomi SJ, Highley JD. 2004 Biobehavioural correlates of hand preference in free-ranging female primates. Laterality **9**, 267-285. (10.1080/13576500342000086) 15341426

[38] Furnham A. 1983 Personality and handedness. Pers. Individ. Dif. **4**, 715-716. (10.1016/0191-8869(83)90132-0)

[39] Reddon AR, Hurd PL. 2008 Aggression, sex and individual differences in cerebral lateralization in a cichlid fish. Biol. Lett. **4**, 338-340. (10.1098/rsbl.2008.0206) 18522921 PMC2610155

[40] Huber KB, Marsolek CJ. 2022 Do cerebral motivational asymmetries mediate the relationship between handedness and personality? Laterality **27**, 21-56. (10.1080/1357650X.2021.1942483) 34238115

[41] Coren S. 1994 Personality differences betwteen left- and right-handers: an overlooked minority group? J. Res. Pers. **28**, 214-229. (10.1006/jrpe.1994.1016)

[42] Killgore WDS, Della PL, Casasanto DJ. 1999 Hemispheric laterality and self-rated personality traits. Percept. Mot. Skills **89**, 994-996. (10.2466/pms.1999.89.3.994) 10665036

[43] Box HO. 1977 Observations on spontaneous hand use in the common marmoset (*Callithrix jacchus*). Primates **18**, 395-400. (10.1007/BF02383117)

[44] Hook-Costigan MA, Rogers LJ. 1995 Hand, mouth and eye preference in the common marmoset (*Callithrix jacchus*). Folia Primatol. **64**, 180-191. (10.1159/000156851) 8613126

[45] Rothe H. 1973 Handedness in the common marmoset (*Callithrix jacchus*). Am. J. Phys. Anthropol. **38**, 561-565. (10.1002/ajpa.1330380267) 4632104

[46] Cameron R, Rogers LJ. 1999 Hand preference of the common marmoset (*Callithrix jacchus*): problem solving and responses in a novel setting. J. Comp. Psychol. **113**, 149-157. (10.1037/0735-7036.113.2.149)

[47] Malukiewicz J et al. 2020 An introduction to the callithrix genus and overview of recent advances in marmoset research. ILAR J. **61**, 110-138. (10.1093/ilar/ilab027) 34933341

[48] Masilkova M, Boukal D, Ash H, Buchanan-Smith HM, Konečná M. 2022 Linking personality traits and reproductive success in common marmoset (*Callithrix jacchus*). Sci. Rep. **12**, 13341. (10.1038/s41598-022-16339-4) 35922528 PMC9349211

[49] Hook MA, Rogers LJ. 2000 Development of hand preferences in marmosets (*Callithrix jacchus*) and effects of aging. J. Comp. Psychol. **114**, 263-271. (10.1037/0735-7036.114.3.263) 10994842

[50] Hook-Costigan MA, Rogers LJ. 1998 Eye preferences in common marmosets (*Callithrix jacchus*): influence of age, stimulus, and hand preference. Laterality **3**, 109-130. (10.1080/713754297) 15513078

[51] Cordeiro de Sousa MB, Xavier NS, Alves da Silva HP, De Oliveira MS, Yamamoto ME. 2001 Hand preference study in marmosets (*Callithrix jacchus*) using food reaching tests. Primates **42**, 57-66. (10.1007/BF02640689)

[52] Hashimoto T, Yamazaki Y, Iriki A. 2013 Hand preference depends on posture in common marmosets. Behav. Brain Res. **248**, 144-1150. (10.1016/j.bbr.2013.04.001) 23578761

[53] Vaughan E, Le A, Casey M, Workman KP, Lacreuse A. 2019 Baseline cortisol levels and social behavior differ as a function of handedness in marmosets (*Callithrix jacchus*). Am. J. Primatol. **81**, e23057. (10.1002/ajp.23057) 31566763 PMC9131356

[54] Matoba M, Masataka N, Tanioka Y. 1991 Cross-generational continuity of hand-use preferences in marmosets. Behaviour **117**, 281-286. (10.1163/156853991X00580)

[55] Tomassetti D, Caracciolo S, Manciocco A, Chiarotti F, Vitale A, De Filippis B. 2019 Personality and lateralization in common marmosets (*Callithrix jacchus*). Behav. Process. **167**, 103899. (10.1016/j.beproc.2019.103899) 31326510

[56] Braccini SN, Caine NG. 2009 Hand preference predicts reactions to novel foods and predators in marmosets (*Callithrix geoffroyi*). J. Comp. Psychol. **123**, 18-25. (10.1037/a0013089) 19236141

[57] Gordon DJ, Rogers LJ. 2010 Differences in social and vocal behavior between left- and right-handed common marmosets (*Callithrix jacchus*). J. Comp. Psychol. **124**, 402-411. (10.1037/a0019736) 20853949

[58] Weiss A, Yokoyama C, Hayashi T, Inoue-Murayama M. 2021 Personality, subjective well-being, and the serotonin 1a receptor gene in common marmosets (*Callithrix jacchus*). PLoS ONE **16**, e0238663. (10.1371/journal.pone.0238663) 34370743 PMC8351977

[59] Masilkova M, Weiss A, Šlipogor V, Konečná M. 2020 Comparative assessment of behaviorally derived personality structures in golden-handed Tamarins (*Saguinus midas*), cotton-top tamarins (*Saguinus oedipus*), and common marmosets (*Callithrix jacchus*). J. Comp. Psychol. **134**, 453-466. (10.1037/com0000226) 32391706

[60] Šlipogor V, Gunhold-de Oliveira T, Tadić Z, Massen JJM, Bugnyar T. 2016 Consistent inter-individual differences in common marmosets (*Callithrix jacchus*) in boldness-shyness, stress-activity, and exploration-avoidance. Am. J. Primatol. **78**, 961-973. (10.1002/ajp.22566) 27286098 PMC4996331

[61] Koski SE, Burkart JM. 2015 Common marmosets show social plasticity and group-level similarity in personality. Sci. Rep. **5**, 8878. (10.1038/srep08878) 25743581 PMC5155412

[62] Iwanicki S, Lehmann J. 2015 Behavioral and trait rating assessments of personality in common marmosets (*Callithrix jacchus*). J. Comp. Psychol. **129**, 205-217. (10.1037/a0039318) 26075516

[63] Šlipogor V, Massen JJMM, Schiel N, Souto A, Bugnyar T. 2021 Temporal consistency and ecological validity of personality structure in common marmosets (*Callithrix jacchus*): a unifying field and laboratory approach. Am. J. Primatol. **83**, e23229. (10.1002/ajp.23229) 33464603 PMC7900989

[64] Šlipogor V, Burkart JM, Martin JS, Bugnyar T, Koski SE. 2020 Personality method validation in common marmosets (*Callithrix jacchus*): getting the best of both worlds. J. Comp. Psychol. **134**, 52-70. (10.1037/com0000188) 31328951

[65] Masilkova M, Weiss A, Konečná M. 2018 How long does it take? Reliable personality assessment based on common behaviour in cotton-top tamarins (*Saguinus oedipus*). Behav. Process. **157**, 59-67. (10.1016/j.beproc.2018.08.009) 30157466

[66] Martin P, Bateson P. 2007 Measuring behaviour: an introductory guide, 3rd edn. Cambridge, UK: Cambridge University Press.

[67] Shannon CE, Weaver W. 1963 The mathematical theory of communication. Urbana, IL: University of Illinois Press.

[68] R Core Team 2020 R: A language and environment for statistical computing. Vienna, Austria: R Foundation for Statistical Computing. https://www.R-project.org/.

[69] Gamer M, Lemon J, Fellows I, Singh P. 2019 Various Coefficients of Interrater Reliability and Agreement. R Package version 0.84.1.

[70] Nakagawa S, Schielzeth H. 2010 Repeatability for Gaussian and non-Gaussian data: a practical guide for biologists. Biol. Rev. **85**, 935-956. (10.1111/j.1469-185X.2010.00141.x) 20569253

[71] Stoffel M, Nakagawa S, Schielzeth H. 2019 rptR: repeatability estimation for Gaussian and Non-Gaussian Data. R Package version 0.9.22.

[72] Revelle W. 2021 Procedures for Psychological, Psychometric and Personality Research. *R Package version 2.2.5*.

[73] Horn JL. 1965 A rationale and test for the number of factors in factor analysis. Psychometrika **30**, 179-185. (10.1007/BF02289447) 14306381

[74] Morton FB, Altschul D. 2019 Data reduction analyses of animal behaviour: avoiding Kaiser's criterion and adopting more robust automated methods. Anim. Behav. **149**, 89-95. (10.1016/j.anbehav.2019.01.003)

[75] Dinno A. 2018 paran: Horn's test of principal components/factors. *R Package version 1.5.2*.

[76] Wilson VAD, Inoue-Murayama M, Weiss A. 2018 A comparison of personality in the common and bolivian squirrel monkey (*Saimiri sciureus* and *Saimiri boliviensis*). J. Comp. Psychol. **132**, 24-39. (10.1037/com0000093) 29239646

[77] Manson JH, Perry S. 2013 Personality structure, sex differences, and temporal change and stability in wild white-faced capuchins, *Cebus capucinus*. J. Comp. Psychol. **127**, 299-311. (10.1037/a0031316) 23339561

[78] Gorsuch RL. 1983 Factor analysis, 2nd edn. Hillsdale, NJ: Lawrence Erlbaum Associates.

[79] Budaev S V. 2010 Using principal components and factor analysis in animal behaviour research: caveats and guidelines. Ethology **116**, 472-480. (10.1111/j.1439-0310.2010.01758.x)

[80] Kaiser HF. 1974 An index of factorial simplicity. Psychometrika **39**, 31-36. (10.1007/BF02291575)

[81] Bartlett MS. 1954 A note on the multiplying factors for various *χ*2 approximations. J. R. Stat. Soc. Ser. B **16**, 296-298. (10.1111/j.2517-6161.1954.tb00174.x)

[82] Koski SE, Buchanan-Smith HM, Burkart JM, Bugnyar T, Weiss A. 2017 Common marmoset (*Callithrix jacchus*) personality. J. Comp. Psychol. **131**, 326-336. (10.1037/com0000089) 29022726

[83] Inoue-Murayama M, Yokoyama C, Yamanashi Y, Weiss A. 2018 Common marmoset (*Callithrix jacchus*) personality, subjective well-being, hair cortisol level and AVPR1a, OPRM1, and DAT genotypes. Sci. Rep. **8**, 10255. (10.1038/s41598-018-28112-7) 29980755 PMC6035208

[84] Epple G. 1970 Quantitative studies on scent marking in the marmoset (*Callithrix jacchus*). Folia Primatol. **13**, 48-62. (10.1159/000155308) 4988037

[85] Barros M, Boere V, Huston JP, Tomaz C. 2000 Measuring fear and anxiety in the marmoset (*Callithrix penicillata*) with a novel predator confrontation model: effects of diazepam. Behav. Brain Res. **108**, 205-211. (10.1016/S0166-4328(99)00153-9) 10701664

[86] Kaplan G, Pines MK, Rogers LJ. 2012 Stress and stress reduction in common marmosets. Appl. Anim. Behav. Sci. **137**, 175-182. (10.1016/j.applanim.2011.04.011)

[87] King JE, Weiss A, Sisco MM. 2008 Aping humans: age and sex effects in chimpanzee (*Pan troglodytes*) and human (*Homo sapiens*) personality. J. Comp. Psychol. **122**, 418-427. (10.1037/a0013125) 19014265

[88] Chopik WJ, Kitayama S. 2018 Personality change across the life span: insights from a cross-cultural, longitudinal study. J. Pers. **86**, 508-521. (10.1111/JOPY.12332) 28646503 PMC5742083

[89] Donnellan MB, Lucas RE. 2008 Age differences in the big five across the life span: evidence from two national samples. Psychol. Aging **23**, 558-566. (10.1037/a0012897) 18808245 PMC2562318

[90] Kuhar CW, Stoinski TS, Lukas KE, Maple TL. 2006 Gorilla behavior index revisited: age, housing and behavior. Appl. Anim. Behav. Sci. **96**, 315-326. (10.1016/j.applanim.2005.06.004)

[91] Seyfarth RM, Silk JB, Cheney DL. 2012 Variation in personality and fitness in wild female baboons. PNAS **109**, 16 980-16 985. (10.1073/pnas.1210780109) PMC3479518 23027933

[92] Silk JB, Beehner JC, Bergman TJ, Crockford C, Engh AL, Moscovice LR, Wittig RM, Seyfarth RM, Cheney DL. 2010 Strong and consistent social bonds enhance the longevity of female baboons. Curr. Biol. **20**, 1359-1361. (10.1016/j.cub.2010.05.067) 20598541

[93] Weiss A, King JE. 2015 Great ape origins of personality maturation and sex differences: a study of orangutans and chimpanzees. J. Pers. Soc. Psychol. **108**, 648-664. (10.1037/pspp0000022) 25402680

[94] Harrison LM, Noble DWA, Jennions MD. 2021 A meta-analysis of sex differences in animal personality: no evidence for the greater male variability hypothesis. Biol. Rev. **97**, 679-707. (10.1111/brv.12818) 34908228

[95] Šlipogor V, Graf C, Massen JJM, Bugnyar T. 2022 Personality and social environment predict cognitive performance in common marmosets (*Callithrix jacchus*). Sci. Rep. **12**, 6702. (10.1038/s41598-022-10296-8) 35513400 PMC9072541

[96] Hook MA, Rogers LJ. 2008 Visuospatial reaching preferences of common marmosets (*Callithrix jacchus*): an assessment of individual biases across a variety of tasks. J. Comp. Psychol. **122**, 41-51. (10.1037/0735-7036.122.1.41) 18298280

[97] Díaz S, Murray L, Roberts SGB, Rodway P. 2021 Between-task consistency, temporal stability and the role of posture in simple reach and fishing hand preference in chimpanzees (*Pan troglodytes*). Appl. Anim. Behav. Sci. **242**, 105417. (10.1016/j.applanim.2021.105417)

[98] Masilkova M, Šlipogor V, Lima Marques Silva GH, Hadová M, Lhota S, Bugnyar T, Konečná M. 2022 Age, but not hand preference, is related to personality traits in common marmosets (*Callithrix jacchus*). *Figshare*. (10.6084/m9.figshare.c.6249011) PMC9579762 36300134

